# Immunocytochemical assessment of sigma-1 receptor and human sterol isomerase in breast cancer and their relationship with a series of prognostic factors

**DOI:** 10.1054/bjoc.2000.1162

**Published:** 2000-05-18

**Authors:** J Simony-Lafontaine, M Esslimani, E Bribes, S Gourgou, N Lequeux, R Lavail, J Grenier, A Kramar, P Casellas

**Affiliations:** Departments of PathologyBiostatistics, Nuclear Medicine, Montpellier Cancer Institute, Montpellier, France; Immuno-Oncology Department, Sanofi-Synthelabo, 371 rue du Prof. Joseph Blayac, Montpellier Cedex 04, 34184, France

**Keywords:** sigma-1 receptor, human sterol isomerase, SR-BP-1, immunocytochemistry, breast cancer, prognostic factors

## Abstract

The purpose of this study was to immunocytochemically investigate two new markers, the sigma-1 receptor and the human sterol isomerase (hSI), in comparison with a series of clinicopathological and immunocytochemical prognostic factors in a trial including 95 patients with operable primary breast cancers. Our results showed no statistically significant relationship between these two markers and the age of the patients, their menopausal status, the tumour size and its histological grade, the nodal status and the expression of the Ki-67 proliferative marker. However, we evidenced a close correlation between the sigma-1 receptor expression and the hormonal receptor positivity (*P* = 0.008), essentially due to a link with the progesterone receptor status (*P* = 0.01). By contrast there was an inverse relationship between hSI expression and the oestrogen receptor and/or progesterone receptor positivity (*P* = 0.098). A significant relationship was shown between both the sigma-1 receptor, hSI expressions and Bcl2 expression, with *P* = 0.017 and 0.035 respectively. We also assessed whether the expression of the sigma-1 receptor or hSI might be linked with disease-free survival (DFS) and found that the presence of hSI and the absence of sigma-1 receptor expression were associated with a poorer disease-free survival (*P* = 0.007). Altogether these results suggest that in primary breast carcinomas in association with the evaluation of the steroid receptor status, the sigma-1 receptor and hSI may be interesting new markers useful to identify those patients who might be able to benefit from an adjuvant therapy. © 2000 Cancer Research Campaign


*British Journal of Cancer* (2000) **82**(12), 1958–1966
© 2000 Cancer Research Campaign
doi: 10.1054/ bjoc.2000.1162, available online at http://www.idealibrary.com on IDEAL®

### Immunocytochemical assessment of sigma-1 receptor and human sterol isomerase in breast cancer and their relationship with a series of prognostic factors

**J Simony-Lafontaine[1], M Esslimani[1], E Bribes[4], S Gourgou[2], N Lequeux[1], R Lavail[1], J Grenier[3], A Kramar[2] and P Casellas[4]**

Departments of [1]Pathology, [2]Biostatistics and [3]Nuclear Medicine, Montpellier Cancer Institute, Montpellier, France; [4]Sanofi-Synthelabo, Immuno-Oncology Department, 371 rue du Prof. Joseph Blayac, 34184 Montpellier Cedex 04, France

**Summary** The purpose of this study was to immunocytochemically investigate two new markers, the sigma-1 receptor and the human sterol isomerase (hSI), in comparison with a series of clinicopathological and immunocytochemical prognostic factors in a trial including 95 patients with operable primary breast cancers. Our results showed no statistically significant relationship between these two markers and the age of the patients, their menopausal status, the tumour size and its histological grade, the nodal status and the expression of the Ki-67 proliferative marker. However, we evidenced a close correlation between the sigma-1 receptor expression and the hormonal receptor positivity ($P$ = 0.008), essentially due to a link with the progesterone receptor status ($P$ = 0.01). By contrast there was an inverse relationship between hSI expression and the oestrogen receptor and/or progesterone receptor positivity ($P$ = 0.098). A significant relationship was shown between both the sigma-1 receptor, hSI expressions and Bcl2 expression, with $P$ = 0.017 and 0.035 respectively. We also assessed whether the expression of the sigma-1 receptor or hSI might be linked with disease-free survival (DFS) and found that the presence of hSI and the absence of sigma-1 receptor expression were associated with a poorer disease-free survival ($P$ = 0.007). Altogether these results suggest that in primary breast carcinomas in association with the evaluation of the steroid receptor status, the sigma-1 receptor and hSI may be interesting new markers useful to identify those patients who might be able to benefit from an adjuvant therapy. © 2000 Cancer Research Campaign

**Keywords**: sigma-1 receptor; human sterol isomerase; SR-BP-1; immunocytochemistry; breast cancer; prognostic factors

Appropriate parameters for predicting the aggressiveness of tumours and their sensitivity to treatment are crucial in cancer therapy: reliable prognostic factors are needed to select the optimum treatment and the follow-up strategies. In breast cancer, the lymph node status is currently one of the best prognostic factors but alone it is not sufficiently accurate to predict the clinical course of the disease (Mink et al, 1994; Hawkins et al, 1996). In addition to this classical morphological prognosis factor of breast carcinoma, many other immunohistochemical markers of different value exist. They are used to predict the clinical course of breast cancer at the time of primary treatment, their evaluation made it possible to offer adjuvant therapy (cytotoxic or endocrine) for patients with a poor prognosis. In that case, oestrogen and progesterone status of primary breast tumours have been shown closely correlated with the therapeutic response to endocrine therapy (Hawkins et al, 1996; Pichon et al, 1996; Robertson et al, 1996). Although the repertoire of the predictive factors contains many different markers characterized so far, their optimal combination remain elusive.

Recently we have characterized two new markers related to cell proliferation, i.e. SR31747 binding protein (SR-BP-1) and the human sterol isomerase (hSI) (Silve et al, 1996; Jbilo et al, 1997). SR-BP-1 was found to be identical to the sigma-1 receptor, and hSI identical to emopamil binding protein (EBP). These two proteins show interesting properties: (1) both proteins are co-localized and their expression was observed to be associated with the endoplasmic reticulum and with the nuclear envelope; (2) these two proteins bind SR31747 with very similar high affinities highlighting the remarkable functional homology between these two SR31747 receptors (Dussossoy et al, 1999).

The SR31747 molecule is a novel agent that elicits immunosuppressive and anti-inflammatory effects. SR31747 has also been shown to block the proliferation of lymphocytes (Casellas et al, 1994) as well as tumour cells (Labit-Le Bouteiller et al, 1998). Recently we reported that the binding of SR31747 on hSI was efficiently inhibited by the tamoxifen molecule with an $IC_{50}$ value in the nanomolar range (Paul et al, 1998). Tamoxifen is a tryphenyl ethylene type of non-steroidal anti-oestrogen. It is being widely used as a therapeutic agent in oestrogen-dependent tumour therapy, specially in breast cancer. In addition to bind to the oestrogen receptor (ER) with high affinity, tamoxifen also binds to sites localized in the AEBS cell microsomal fraction (anti-oestrogen binding site). We have shown that the AEBS is the EBP (hSI) (Paul et al, 1998).

Altogether these data made it interesting to test whether the sigma-1 receptor and hSI would be significant markers for prognostic purposes in breast cancers. To assess their prognostic significance, we studied their immunohistochemical expression in primary invasive breast cancers of pre- and post-menauposal patients. In addition, we investigated their relationship with well

*Received 28 April 1999*
*Accepted 17 January 2000*

*Correspondence to:* P Casellas established prognostic factors, including standard histological criteria (tumour grading, size, nodal status, etc.), immunohistochemical markers of cell proliferation (Ki-67, MIB-1), and cell death using the Bcl2 proto-oncogene whose over-expression has been shown generally associated with ER-positive status and often with a favourable prognosis (Gee et al, 1994; Johnston et al, 1994; Hellemans et al, 1995; Buckholm et al, 1997; Slooten et al, 1998).

### MATERIALS AND METHODS

### Patients

From January 1992 to February 1993, 850 new breast cancers were diagnosed at the CRLC Department of Pathology (Montpellier, France). Selection criteria included presentation with primary invasive breast carcinoma, no preoperative chemotherapy, endocrine therapy or radiation therapy, sufficient tumour tissue remaining after diagnosis to allow biochemical quantification of receptors status and additional immunohistochemical assays (practically tumour size more than 1-cm diameter), and long-term follow-up for disease recurrence and death. A total of 95 patients who satisfied these criteria were chosen.

Surgical treatment included radical mastectomy with axillary dissection in 58% of the patients and breast conservative sector resection with axillary dissection in 42% of the patients. After surgery, all the patients with conservative treatment and 60% with radical mastectomy underwent combined post-operative radiotherapy, to eradicate local remainders of the disease. Eighty per cent received systemic adjuvant therapy, according to the CRLC routinely assessed clinical management of the disease, and depending on their age, menopausal status, steroid receptor status and nodal status: chemotherapy alone for 16 patients, endocrine therapy alone (tamoxifen) for 58 patients, combined chemotherapy and endocrine therapy for two patients. Patients were observed for disease recurrence and death, with a mean follow-up of 64 months.

### Tumour samples

At surgery, all patients had a small portion of the tumour removed which was snap-frozen in liquid nitrogen and stored at –80°C for ER and progesterone receptor (PR) analysis. The remaining part of the tumour was fixed in formalin-alcohol for 24 h, parrafin embedded and subsequently processes with routine techniques followed by immunohistochemical analysis.

**Table 1** Antibodies used in this study

| Antigen | Source | Pretreatment antibody dilution |
|---|---|---|
| SR-BP-1 | Mouse monoclonal antibody, | NT |
| Sigma-1 receptor | Sanofi-Synthelabo | 1/400 |
| hSI | Rabbit polyclonal antibody, | MW |
| Human sterol isomerase | Sanofi-Synthelabo | 1/100 |
| Bcl2 | Mouse monoclonal antibody, | MW |
| | Clone 124 | 1/50 |
| | Dako A/S, Denmark | |
| Ki-67 | MIB-1, | MW |
| | Immunotech, France | 1/100 |

MW: microwave epitope retrieval; NT: no pretreatment

### Histopathological study

Five-micron-thick tumour slides were stained with haematoxylin and eosin for the histopathological study. Tumour grading was performed according to the methodology of Scarff et al (1957), modified by Elston et al (1987, 1991). Mitosis counts were performed in ten high-power fields (HPF = 400 ×) using a Leica microscope (Leitz DMRB). The tumour size was recorded as the maximum diameter of the surgically-removed tumour mass. Axillary lymph node status was assessed for each case by histopathological examination for a minimum of seven lymph nodes.

### Immunohistochemical analysis

The expressions of the sigma-1 receptor, hSI, Bcl2 and the proliferative marker Ki-67 were analysed using an immunohistochemical procedure. The antibodies used were: a mouse monoclonal anti-sigma-1 receptor (Jbilo et al, 1997), a rabbit polyclonal anti-hSI raised against the N-terminal (2–25) peptide of the hSI (its specificity was assessed using the competitive immunogene peptide as a reference) (Dussossoy et al, 1999), a mouse monoclonal anti-Bcl2 antibody (Dako, clone 124) and the anti-Ki-67 MIB-1 antibody (Immunotech). Their respective dilution used were 1:400, 1:100, 1:50 and 1:100. These four antibody characteristics (sources, dilutions) are summarized in Table 1. Two-micrometre-thick paraffin-embedded sections of tumour samples were analysed, mounted on Dako silanized slides. All procedures were carried out at room temperature. Immunohistochemical detection of the different markers was done using the streptavidin–biotin (LSAB) method (Dako LSAB kit). The sections, which had been preincubated with 3% hydrogen peroxide ($H_2O_2$) solution for 10 min to block endogenous peroxidase, were incubated for 20 min with blocking agent, for 2 h with the different primary antibodies, they were then rinsed and incubated with the secondary antibody for 10 min. They were then incubated with streptavidin conjugated to horseradish peroxidase: a positive reaction was visualized with 3-amino-9-ethylcarbazol. Before mounting, the sections were counterstained with Mayer’s haematoxylin. For the negative control, the primary antibody was omitted and replaced by an irrelevant antibody (monoclonal mouse anti-human IgG (Dako)). For the positive control, sections from normal breast tissue were used.

The different marker’s immunoreactivity was then evaluated by two observers using a high-power lense (400 ×). Cytoplasmic (sigma-1 receptor, hSI, Bcl2) and nuclear (MIB-1) labelling were evaluated using a semiquantitative method taking into account the staining intensity and the number of stained cells in different random fields: 0 means no staining or less than 10% tumour cells labelled, 1 means a weak staining from 10 to 30% tumour cells, 2 means a moderate staining in more than 30% tumour cells, and 3 means an intense and diffuse staining.

### Quantification of steroid hormone receptors

Breast tumour specimens were frozen in liquid nitrogen immediately after surgical removal and send to the Steroid Receptor Laboratory, then they were pulverized in liquid nitrogen, cytosols were prepared and the dextran-coated charcoal assay was used to determine the receptor status with $^3H$ oestradiol and $^3H$ progesterone as labelled ligands. The results were expressed as

© 2000 Cancer Research Campaign 

fentomols per milligram of tissue (fmol $mg^{-1}$). Values greater than 10 fmol $mg^{-1}$ were considered as positive.

### Statistical analysis

Correlations between the clinico-pathological data and the expression of the four immunohistochemical markers analysed were assessed using standard $\chi^2$ tests. The median values of different variables were compared using the Kruskal–Wallis test. Locoregional disease relapse and/or distant metastasis and death due to cancer were considered as end points for disease-free survival (DFS). DFS curves starting from the date of surgery were plotted using the Kaplan–Meier method. The statistical significance of each marker was calculated using the log-rank test. For all statistical analyses, a $P$-value < 0.05 was considered statistically significant. $P$-values over 0.10 are noted 'NS' for non significant. For further statistical analysis, two groups of patients were defined: patients who underwent adjuvant endocrine therapy (tamoxifen) and total population, including patients with or without adjuvant therapy (endocrine or chemotherapy).

### RESULTS

### Patient characteristics (Table 2)

The analysis of the four markers under study is performed with 95 patients. Patients were characterized according to their age, their menopausal status (assessed using serum gonadotrophin, oestradiol and progesterone measurements in pre and peri-menopausal patients), the size of the tumour, the axillary nodal status, the TNM staging (based on the UICC Atlas criteria, 1992) and the therapy type. The mean age of the patients was 61 years (range 32–83); 79% of the patients were post-menopausal. Among the pre-menopausal patients, 60% were younger than 45 years. Within this population, eight patients had recurrences (locoregional, three; controlateral, five) after a mean time of 38 months (range 23–64), 18 patients had distant metastasis after a mean time of 44 months (range 23–73). The number of deaths was four after a mean time of 44 months (range 41–72).

**Table 2** Population characteristics

| Features | Number of patients | % |
|---|---|---|
| Total population | 95 | |
| *Population characteristics* | | |
| Age (years) | | |
| Median | 61 | |
| Range (min–max) | 32–83 | |
| Menopause | | |
| Pre- | 20 | 21 |
| Post- | 75 | 79 |
| *Therapeutic characteristics* | | |
| Adjuvant therapy | | |
| None | 19 | 18 |
| Endocrine therapy | 58 | 62 |
| Chemotherapy | 18 | 20 |
| *Disease status* | | |
| Tumour grade | | |
| I | 22 | 24 |
| II | 38 | 40 |
| III | 32 | 33 |
| No grading | 3[a] | 3 |
| Tumour size | | |
| T1 | 54 | 57 |
| T2 | 37 | 39 |
| T3 | 4 | 4 |
| Nodal status | | |
| N0 | 55 | 58 |
| N1 | 40 | 42 |
| TNM stage | | |
| I | 37 | 38 |
| IIA | 34 | 35 |
| IIB | 21 | 22 |
| IIIA | 3 | 5 |
| *Steroid receptor status* | | |
| ER status | | |
| Positive | 62 | 65 |
| Negative | 33 | 35 |
| PR status | | |
| Positive | 66 | 69 |
| Negative | 29 | 31 |
| ER median (range) | 59 (10–441) | |
| PR median (range) | 76 (10–576) | |

[a] Breast carcinomas of special types.

### Histopathological findings (Table 2)

Clinical tumour size was less than 20 mm in 57% of cases (T1), between 20 and 50 mm in 39% (T2), and more than 50 mm in 4% (T3). Eighty-five per cent of the cancers were of infiltrating ductal type, 11% were of infiltrating lobular type, 4% were of other types. According to the Elston and Ellis modification of the Bloom and Richardson grading system (SBR), 24% of patients were grade I, 41% grade II and 35% grade III. Forty-two per cent of the patients were axillary lymph node-positive, the other being lymph node-negative.

### Steroid receptor status

ER-positive status was observed in 65% of the tumours with a median concentration of 59 fmol $mg^{-1}$ for ER-positive patients (range 10–441 fmol $mg^{-1}$). Sixty-nine per cent of the tumours were PR-positive with a median concentration of 76 fmol $mg^{-1}$ (range 10–576 fmol $mg^{-1}$). Positivity for both receptors was observed in 55% of the tumours (52 cases). Only one of the receptors (ER or PR) was positive in 25% of the tumours (24 cases), and ER and PR were both negative in 19 cases (20%).

### Immunohistochemical findings

### *Immunohistochemical distribution of the sigma-1 receptor expression (Figure 1 A, B)*

The sigma-1 receptor was present in normal breast sections, heterogeneously distributed in epithelial ducts and acinar structures, and the immunostaining was never strong (Figure 1B). Positive cells showed a cytoplasmic granular staining, very often with a perinuclear localization. Metaplastic apocrine epithelial cells of microcyst structures were also stained. In addition to the epithelial component, several other structures showed weak immunostaining, particularly the smooth muscle cells of vascular sections, the myofibroblastic cells of the stromareaction, and a few histiocytic and mononuclear inflammatory cells. Positive imunostaining for sigma-1 receptor was observed in 72 tumours (76%). Positively stained tumour cells appeared to be homogeneously and strongly stained. The immunostaining was cytoplasmic but the

 © 2000 Cancer Research Campaign

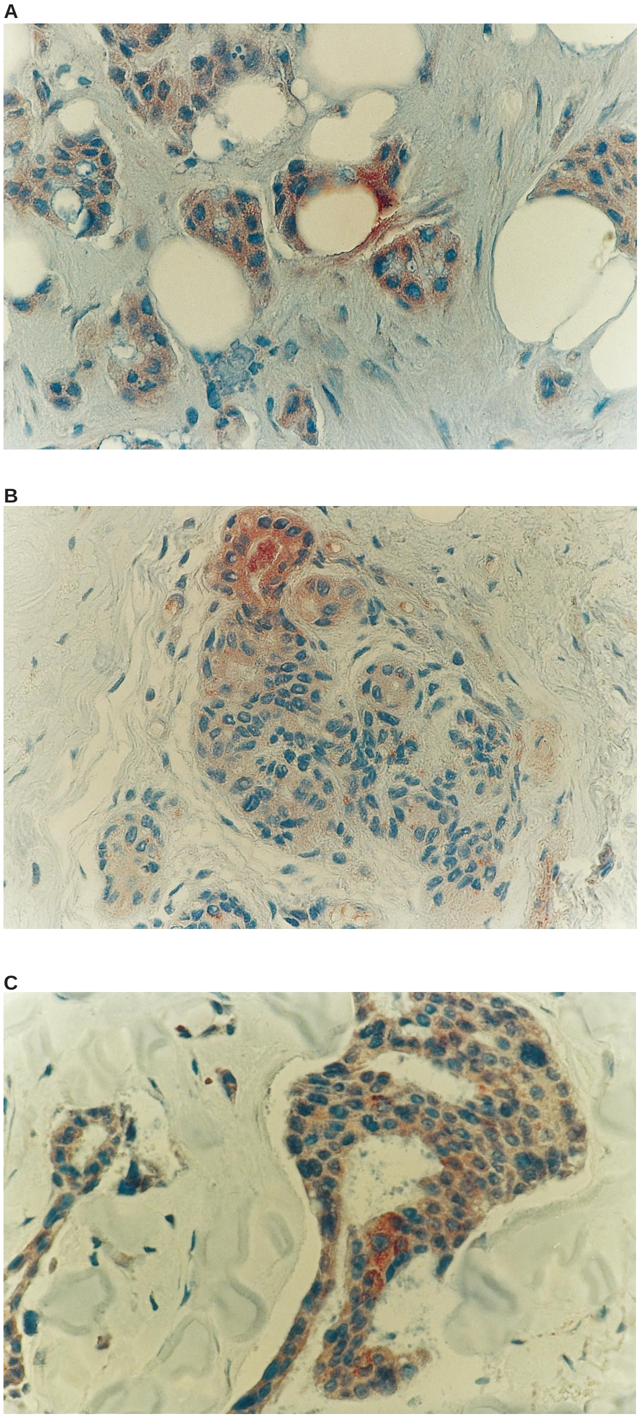

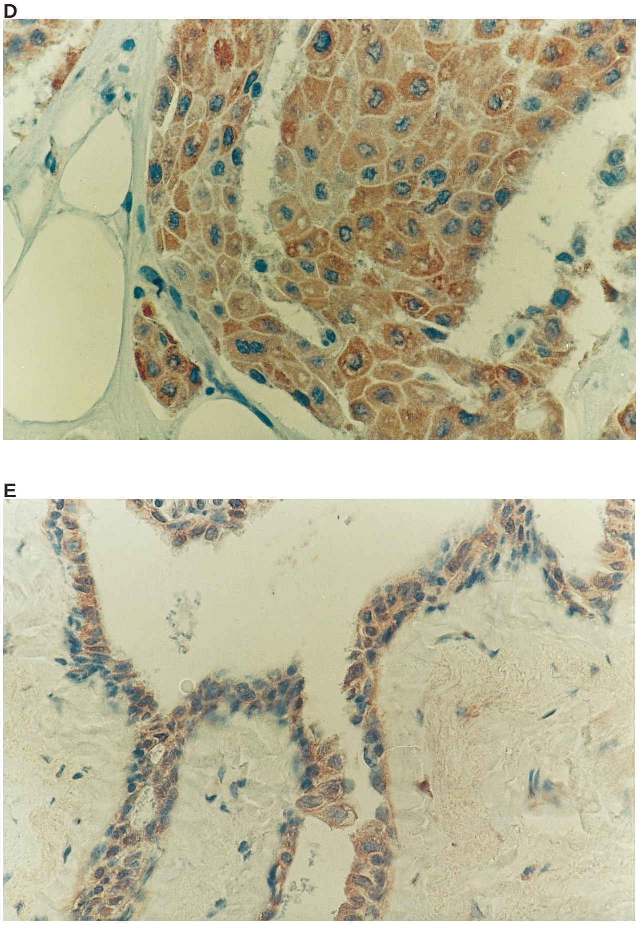

**Figure 1** Immunostaining of (**A**) infiltrating breast cancer or (**B**) normal breast acini with anti-sigma-1 receptor antibody; magnification is × 200. Immunostaining of (**C**) infiltrating breast cancer or (**E**) normal breast duct with anti-hSI antibody; magnification is × 200. A zoom (**D**, magnification is × 400) showed a strong immunostaining of infiltrating breast cancer with anti-hSI antibody with an increase along the inner border of the cell granular and perinuclear pattern seemed to be less obvious (Figure 1A). The intraductal component of the infiltrating cancers generally did not show any staining, or only a weak one.

### *Immunohistochemical distribution of the hSI expression (Figure 1 C–E)*

Normal breast components (ductal and acinar epithelial cells) more often showed a weak cytoplasmic immunostaining (Figure 1E). Positive immunostaining for hSI was observed in 65 tumours (68%). The immunostaining of the epithelial tumoural cells was heterogeneous, with variable intensity within the same tumour and between patients. Cytoplasmic elements were stained, including the endoplasmic reticulum and the nuclear envelope, and often with an increase in the staining along the cytoplasmic envelope (Figure 1 C, D). The intraductal components of the tumours showed no or weak staining.

### *Immunohistochemical expression of Bcl2 and Ki-67*

Bcl2 reactivity was observed in 75 patients (79%). The staining was always cytoplasmic (data not shown). MIB-1 anti-Ki-67 antibody nuclear staining was weak for 29 patients (31%), intermediate for 23 patients (24%) and strong for 43 patients (45%).

### *Associations between sigma-1 receptor and hSI expression with clinicopathological variables (Tables 3 and 4)*

No correlation was shown between the sigma-1 receptor expression and the age, tumour grade, tumour size, or nodal status of the patients. However, an absence of detectable sigma-1 receptor expression was most often observed in premenopausal patients

© 2000 Cancer Research Campaign 

**Table 3** Relationship between sigma-1 receptor, hSI expression and clinicopathological parameters in primary operable breast carcinomas

| Factors | Sigma-1 receptor immunoreactivity (0/1,2,3) | | | hSI immunoreactivity (0/1,2,3) | | |
|---|---|---|---|---|---|---|
| | Negative *n* = 23 | Positive *n* = 72 | *P*-value | Negative *n* = 30 | Positive *n* = 65 | *P*-value |
| Age | | | NS | | | NS |
| <45 years | 3 (13%) | 10 (14%) | | 4 (13%) | 9 (14%) | |
| 45–54 | 4 (17%) | 20 (28%) | | 7 (23%) | 17 (26%) | |
| <54 | 16 (70%) | 42 (58%) | | 19 (64%) | 39 (60%) | |
| Menopausal status | | | 0.09 | | | NS |
| Non- | 2 (9%) | 18 (25%) | | 4 (13%) | 16 (24%) | |
| Post- | 21 (91%) | 54 (75%) | | 26 (87%) | 49 (76%) | |
| Tumour size | | | NS | | | NS |
| T1 | 13 (57%) | 41 (57%) | | 19 (64%) | 35 (54%) | |
| T2 | 9 (39%) | 28 (39%) | | 10 (33%) | 25 (41%) | |
| T3 | 1 (4%) | 3 (4%) | | 1 (3%) | 3 (5%) | |
| Tumour grade | | | NS | | | NS |
| 1 | 3 (14%) | 19 (27%) | | 8 (29%) | 14 (22%) | |
| 2 | 8 (36%) | 30 (43%) | | 11 (39%) | 27 (42%) | |
| 3 | 11 (50%) | 21 (30%) | | 9 (32%) | 23 (36%) | |
| Nodal status | | | NS | | | NS |
| 0 | 11 (48%) | 44 (61%) | | 18 (60%) | 33 (54%) | |
| 1 | 12 (52%) | 28 (39%) | | 12 (40%) | 28 (46%) | |

**Table 4** Relationship between global expression of sigma-1 receptor and hSI antibodies with receptor status in primary operable breast carcinomas

| Receptor status | Sigma-1 receptor immunoreactivity (0/1,2,3) | | | hSI immunoreactivity (0/1,2,3) | | |
|---|---|---|---|---|---|---|
| | Negative | Positive | *P*-value | Negative | Positive | *P*-value |
| Association ER, PR | | | 0.034 | | | 0.027 |
| ER–, PR– | 9 (39%) | 10 (14%) | | 3 (10%) | 16 (36%) | |
| ER–, PR+ | 1 (4%) | 13 (18%) | | 4 (13%) | 10 (23%) | |
| ER+, PR– | 3 (13%) | 7 (10%) | | 5 (17%) | 5 (11%) | |
| ER+, PR+ | 10 (44%) | 42 (58%) | | 18 (60%) | 18 (40%) | |
| Association ER, PR: | | | 0.008 | | | 0.098 |
| ER–, PR– | 9 (39%) | 10 (14%) | | 3 (10%) | 16 (25%) | |
| ER+ or PR+ | 14 (61%) | 62 (86%) | | 27 (90%) | 49 (75%) | |
| ER: | | | | | | |
| Status:– | 10 (44%) | 23 (32%) | NS | 7 (23%) | 26 (40%) | 0.11 |
| + | 13 (56%) | 49 (68%) | | 23 (77%) | 39 (60%) | |
| *values | 119 (13–214) | 55 (10–441) | NS | 40 (10–326) | 102 (11–441) | 0.004 |
| PR: | | | | | | |
| Status:– | 12 (52%) | 17 (24%) | 0.01 | 8 (27%) | 21 (32%) | NS |
| + | 11 (48%) | 55 (76%) | | 22 (73%) | 44 (68%) | |
| Median (range) | 40 (10–346) | 82 (10–576) | NS | 55 (10–576) | 82 (10–448) | NS |

($P$ = 0.09). There was a significant relationship between the sigma-1 receptor expression and the steroid receptor status ($P$ = 0.03). ER and PR were more often negative in the absence of sigma-1 receptor immunoreactivity (39%) than in its presence (14%) ($P$ = 0.008). There was a significant relationship between the sigma-1 receptor immunoreactivity and PR status. Among PR-negative patients, sigma-1 receptor immunostaining was positive in 59% of patients, whereas among PR-positive patients, sigma-1 receptor immunostaining was positive in 83% of patients ($P$ = 0.01). No correlation was found between sigma-1 receptor immunoreactivity and ER status. There was no correlation between positive sigma-1 receptor expression and receptor levels. For hSI immunoreactivity, there was no relationship with the age, menopausal status, tumour grade, tumour size or nodal status of the patients (Table 3). There was a significant correlation between hSI expression and the steroid receptor status ($P$ = 0.027). An absence of immunoreactivity was essentially associated with a positive receptor status for ER and/or PR ($P$ = 0.098). There was a non-significant correlation between hSI expression and ER status ($P$ = 0.11), while the absence of hSI immunoreactivity tended to be associated with ER positivity. Nevertheless, among ER-positive patients, the median ER values were significantly greater with hSI immunoreactivity (102 fmol $mg^{-1}$) than with its absence expression (40 fmol $mg^{-1}$) ($P$ = 0.004). There was no significant correlation between PR status and hSI immunoreactivity or between PR values and hSI expression (Table 4). In the tamoxifen-treated subgroup with positive PR status, the median PR value was greater in the group with highly positive hSI immunoreactivity (292 fmol $mg^{-1}$), than in the group without or only slightly positive hSI expression (73 fmol $mg^{-1}$) ($P$ = 0.056).

 *© 2000 Cancer Research Campaign*

*Associations between sigma-1 expression, hSI expression, Bcl2 and Ki-67 immunostaining (Table 5)*

No significant relationship was found between sigma-1 receptor and hSI expressions. There was no significant relationship between the sigma-1 receptor expression, hSI expression and Ki-67 immunostaining. A significant positive relationship was noted between sigma-1 receptor and Bcl2 positivity ($P = 0.017$), and an inverse relationship between hSI and Bcl2 protein expression ($P = 0.035$).

### Prognostic relevance

Compared to other possible clinico-pathological prognostic factors, sigma-1 receptor and hSI expression was associated with DFS.

In our study, there was a positive relationship between the positive sigma-1 immunostaining and DFS (5-year DFS = 81% vs 60%; $P = 0.09$). A significant relationship was found between DFS and the inverse co-expression of sigma-1 and hSI, with more failures occurring among patients who expressed hSI without sigma-1 expression (5-year DFS = 48%; $P = 0.007$; Figure 2). Patients with Bcl2 positivity had a higher DFS rate (81% at 5 years) than patients who did not express Bcl2 (59% at 5 years, $P = 0.004$; Figure 3). Increased DFS was noted when Bcl2 positivity was associated with sigma-1 positivity (DFS = 85% vs 58%; $P = 0.0004$; Figure 4), and the absence of hSI (DFS = 90% vs 70%; $P = 0.061$; Figure 5). These results were more significant in the group of patients who received tamoxifen. There was a close correlation between sigma-1 positivity and longer DFS (DFS = 84% vs 58%; $P = 0.045$; Figure 6). Shorter DFS was observed with a simultaneous absence of sigma-1 receptor expression and positive hSI antibody immunoreactivity (DFS = 45% vs 85%; $P = 0.003$; Figure 7). As compared with Bcl2 expression, sigma-1 and hSI expression suggested that they had a greater effect on DFS. In the group of patients with a loss of Bcl2 protein, DFS was shorter for patients with positive hSI immunoreactivity (DFS = 40%) than for patients with an absence of hSI expression (DFS = 100%), but due to the small number of patients this result was non-significant (Figure 8). Moreover, for patients with positive Bcl2 expression, the positive sigma-1 immunoreactivity improved DFS (5-year DFS = 90% vs 50% for patients with an absence of sigma-1 expression; $P = 0.001$; Figure 9).

### DISCUSSION

This report describes the comparison of two new markers, the sigma-1 receptor and hSI, with other clinico-pathological prognostic factors in a group of 95 patients with operable primary breast carcinoma. Correlations between the expression of these new markers with the age, menopausal status, tumour size, its nodal status and the steroid receptor status were assessed. Their

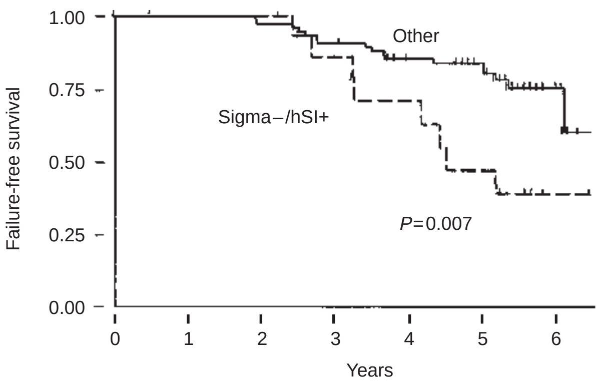

**Figure 2** Disease-free survival: sigma-1-negative/hSI-positive patients versus other all patients

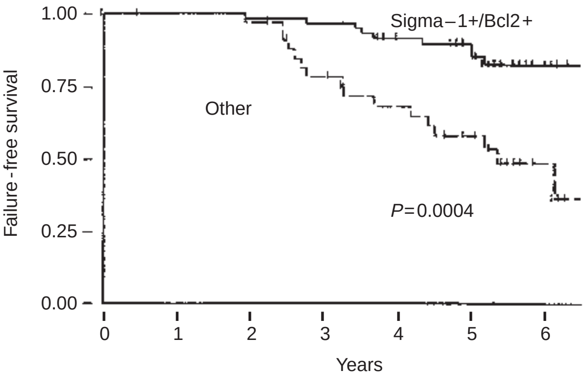

**Figure 4** Disease-free survival: Bcl2-positive/sigma-1-negative versus all other patients

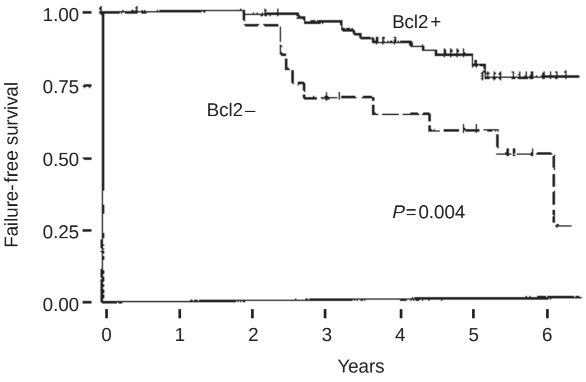

**Figure 3** Disease-free survival: patients were dichotomized as being Bcl2-positive or Bcl2-negative

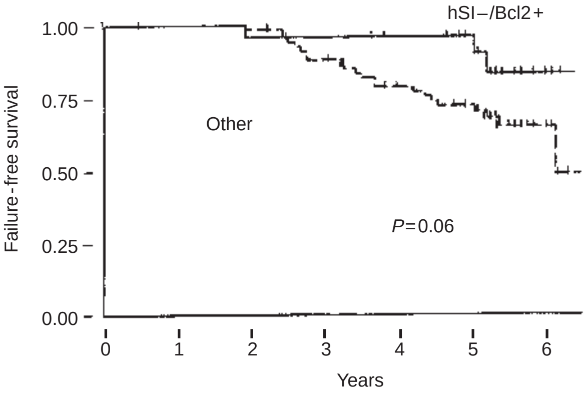

**Figure 5** Disease-free survival: Bcl2-positive/hSI-negative versus all other patients

© 2000 Cancer Research Campaign

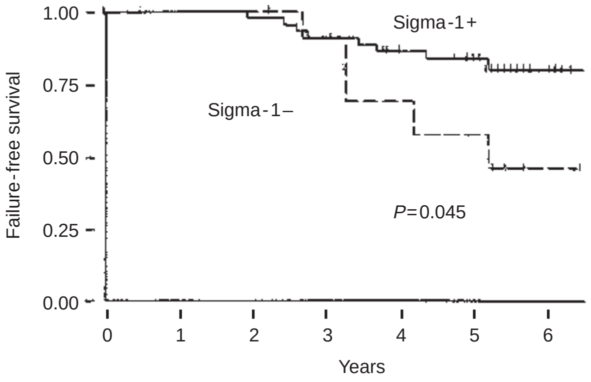

**Figure 6** Disease-free survival: sigma-1-positive versus sigma-1-negative in the tamoxifen-treated group

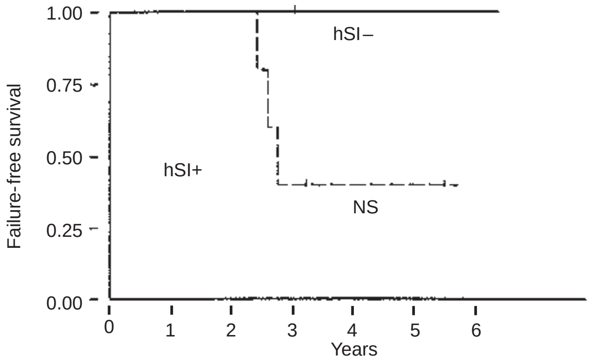

**Figure 8** Disease-free survival: sigma-1-negative/hSI-positive versus all other patients in the tamoxifen-treated group and Bcl2-negative group

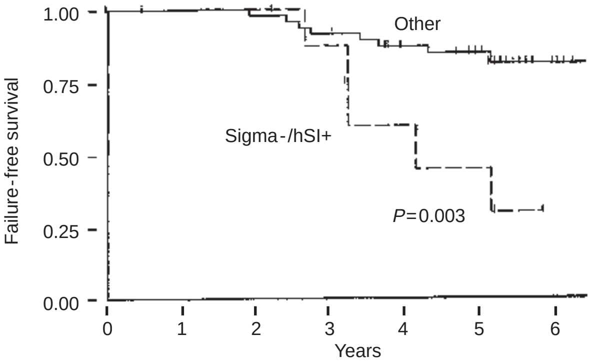

**Figure 7** Disease-free survival: sigma-1-negative/hSI-positive versus all other patients in the tamoxifen-treated group

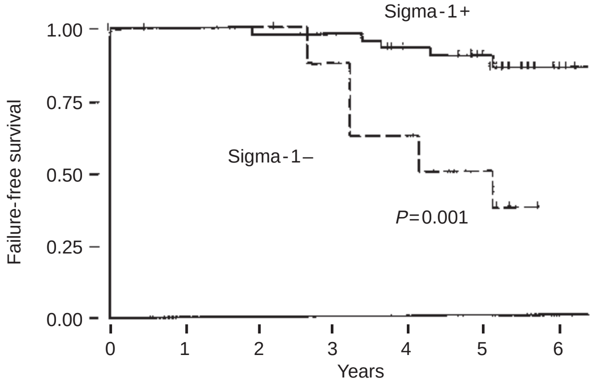

**Figure 9** Disease-free survival: sigma-1-negative/hSI-positive versus all other patients in the tamoxifen-treated group and Bcl2-positive group

**Table 5** Overall correlations between the immunohistochemical markers

| | Sigma1 receptor expression | | | hSI expression | | |
|---|---|---|---|---|---|---|
| | **Negative** | **Positive** | ***P*-value** | **Negative** | **Positive** | ***P*-value** |
| hSI | | | | | | |
| Negative | 7 (30%) | 23 (32%) | NS | – | – | – |
| Positive | 16 (70%) | 49 (68%) | | – | – | |
| Sigma-1 | | | | | | |
| Negative | – | – | – | 7 (23.3%) | 16 (24.6%) | NS |
| Positive | – | – | | 23 (76.7%) | 49 (75.4%) | |
| Bcl2 | | | | | | |
| Negative | 9 (39%) | 11 (15%) | 0.017 | 5 (17%) | 15 (23%) | 0.035 |
| + | 5 (22%) | 10 (14%) | | 1 (3%) | 14 (22%) | |
| ++/+++ | 9 (39%) | 51 (71%) | | 24 (80%) | 36 (55%) | |
| MIB-1 | | | | | | |
| 1 | 6 (26%) | 23 (32%) | | 9 (30%) | 20 (31%) | |
| 2 | 5 (22%) | 18 (25%) | NS | 10 (33%) | 13 (15%) | NS |
| 3 | 12 (52%) | 31 (43%) | | 11 (37) | 32 (54%) | |

relationships with the DFS, the tumour proliferative rate (Ki-67) and with the expression of the Bcl2 proto-oncogene were also investigated. We studied the sigma-1 receptor and hSI expression by immunohistochemical analysis to address their prognostic value for the patient outcome and their potential modulation in response to endocrine therapy.

A multivariate statistical analysis was conducted and showed that among the classical morphological prognosis factors of breast carcinoma and the other immunohistochemical markers studied, only the SBR grading was correlated with DFS in the total population analysed ($n = 95$). However, the subgroup of the patients who received an adjuvant endocrine therapy ($n = 58$), two other factors:

*© 2000 Cancer Research Campaign*

age and menopausal status of the patients were also significantly correlated with DFS. Histopathological tumour grading in breast carcinomas, despite its subjectivity, remains an important prognostic factor, as reported in several studies (Hawkins et al, 1996; Pichon et al, 1996).

Receptor status, along with age and menopausal status, were decisive factors for an adjuvant endocrine therapy in the present study. However, this status was not significantly correlated with DFS. Some authors have reported that the receptor status has a prognostic effect within the first 5 years after surgery, but is no longer pertinent after 8 years of follow-up (Colett et al, 1996; Hawkins et al, 1996; Pichon et al, 1996). In the large series of patients reported by Pichon et al, ER and PR status, as compared with established prognostic factors such as TNM and histological grading, were of relatively limited predictive value. Their major interest remains in therapeutic guidance for subgroups of breast cancer patients. These findings are in agreement with our present data. Steroid receptors represented the only biological parameter in this decision. A recent study reported that Bcl2 immunostaining was a more accurate predictor of response than ERs (Gee et al, 1994). The Bcl2 protein in our study group, i.e. in the overall population and in the subgroup of patients treated by adjuvant endocrine therapy, was the strongest predictor of outcome and response to endocrine therapy. It was compared with the two markers investigated.

Our findings showed a significant correlation between the sigma-1 receptor expression and PR status. Sigma-1 expression occurred essentially with PR-positive tumours, whereas hSI expression had a significant inverse correlation with ER status. There was also a close correlation between Bcl2 protein expression with sigma-1 expression and an inverse correlation with hSI expression. A highly significant relationship was noted between the presence or not of Bcl2 protein and the receptor status. Bcl2 over-expression occurred when ER and/or PR were positive, as also previously demonstrated (Gee et al, 1994; Johnston et al, 1994; Hellemans et al, 1995; Bukholm et al, 1997). These interreactions between receptor status, Bcl2 protein and these two new markers suggest a possible relationship between these proteins. Although the endogenous ligands of sigma sites have not yet been clearly identified, Su (1991) suggested that they belong to the steroid family and possibly include progesterone. We thus considered that they could have a biochemical relationship through their ligands. Furthermore, it is interesting to note that the promoter region sequence of sigma-1 receptor contains binding sites for progesterone, suggesting that sigma-1 receptor expression is regulated by this steroid (Seth et al, 1997). The findings of this study are in accordance with this hypothesis. Altogether these data indicate that sigma-1 receptor expression may be functionally linked to the PR status and thus could be an additional parameter in the choice of patients to undergo endocrine therapy.

The functional activities of sigma-1 and hSI have not yet been clearly elucidated. Nevertheless, these proteins are likely involved in the sterol synthesis on the grounds of following data (Silve et al, 1996): (1) the sequence similarity of yeast sterol isomerase and the sigma receptor binding protein; (2) the mammalian EBP, which is very structurally different from sigma receptor binding protein and yeast sterol isomerase, displays sterol isomerase activity when expressed in yeast; (3) the cellular location of these two proteins in the nuclear membrane and in the ER, which also includes other cholesterol synthesis proteins such as HMG-CoA.

The influence of sigma-1 and hSI expression (alone or combined) was also investigated on DFS. They were found to be significantly associated with DFS, and even more so in the subgroup of tamoxifen-treated patients. The loss of hSI expression and the positive sigma-1 expression was associated with the highest DFS rate, while the presence of hSI and the absence of sigma-1 expression was associated with a lower DFS rate.

The combined loss of sigma-1 expression with the presence of hSI expression was strongly associated with the lowest DFS rate. They seemed to have more of an influence on the DFS in the group of tamoxifen-treated patients.

We conclude that sigma-1 and hSI markers in primary breast carcinomas may have an application in the choice of patients who may benefit from an adjuvant endocrine therapy, in association with an evaluation of the steroid receptor status. Adjuvant endocrine therapy could be recommended for patients with positive expression of sigma-1 antibody and loss of hSI expression. In contrast, the group of patients showing a loss of sigma-1 expression associated with positive hSI expression, despite the positive receptor status, did not have a significant improvement in DFS with adjuvant endocrine therapy. This patient group could probably have benefited from a higher DFS with adjuvant chemotherapy. A larger study should be undertaken to confirm these results and test them for the therapeutic management of patients with primary operable breast carcinomas.

### ACKNOWLEDGEMENTS

We thank Ms V Paganesi and Ms M Radal for their excellent technical assistance, and our colleagues Dr P Rouanet, Dr B Saint-Aubert, Dr F Quenet and Dr G Romieu (from the Montpellier Cancer Institute) for supplying clinical data. We also thank G Loison for helpful discussion during the preparation of the manuscript.

### REFERENCES

Bloom HJG and Richardson WW (1957) Histological grading and prognosis in breast cancer. *Br J Cancer* **11**: 359–377

Bukholm IK, Nesland JM, Karesen R, Jacobsen U and Borresen-Dale A-L (1997) Interaction between bcl-2 and p21 (WAF1/CIP1) in breast carcinomas with wild-type p53. *Int J Cancer* **73**: 38–41

Casellas P, Bourrie B, Canat X, Carayon P, Buisson I, Paul R, Breliere JC and Le Fur G (1994) Immunopharmacological profile of SR31747: in vitro and in vivo studies on humoral and cellular responses. *J Neuroimmunol* **52**: 193–203

Chan WK, Poulsom R, Lu QL, Patel K, Gregory W, Fisher CJ and Hanby AM (1993) Bcl-2 expression in invasive mammary carcinoma: correlation with apoptosis, hormone receptors and p53 expression. *J Pathol* **169**: 138

Collett K, Hartveit F, Skjaerven R and Maehle BO (1996) Prognostic role of oestrogen and progesterone receptors in patients with breast cancer: relation to age and lymph node status. *J Clin Pathol* **49**: 920–925

Derocq JM, Bourrie B, Segui M, Le Fur G and Casellas P (1995) In vivo inhibition of endotoxin-induced pro-inflammatory cytokines production by the sigma ligand SR 31747. *J Pharmacol Exp Ther* **272**: 224–230

Dussossoy D, Carayon P, Belugou S, Feraut D, Bord A, Goubet C, Roque C, Vidal H, Combes T, Loison G and Casellas P (1999) Colocalization of sterol isomerase and sigma1 receptor at endoplasmic reticulum and nuclear envelope level. *Eur J Biochem* **263**: 377–386

Elston CW and Ellis IO (1991) Pathological prognostic factors in breast cancer: experience from a large study with long-term follow-up. *Histopathology* **19**: 403–410

Elston CW (1987) The assessment of histological grade in breast cancer. *Aust NZJ Surg* **54**: 11–15

Gee JMW, Robertson JFR, Ellis IO, Willsher P, Maclelland RA, Hoyle HB, Kyme SR, Finlay P, Blamey RW and Nicholson RI (1994) Immunocytochemical localization

© 2000 Cancer Research Campaign 

of Bcl-2 protein in human breast cancers and its relationship to a series of prognostic markers and response to endocrine therapy. *Int J Cancer* **59**: 619–628

Hawkins RA, Tesdale AL, Killen ME, Jack WJL, Chetty U, Dixon JM, Hulme MJ, Prescott RJ, Mcintyre MA and Miller WR (1996) Prospective evaluation of prognostic factors in operable breast cancer. *Br J Cancer* **74**: 1469–1478

Hellemans P, van Dam PA, Weyler J, van Oosterom At, Buytaert P and Marck EV (1995) Prognostic value of Bcl-2 expression in invasive breast cancer. *Br J Cancer* **72**: 354–360

Hockenbery D, Nunez G, Milliman C, Schreiber RD and Korsmeyer SJ (1990) Bcl-2 is an inner mitochondrial membrane protein that blocks programmed cell death. *Nature* **348**: 334–336

Jbilo O, Vidal H, Paul R, De Nys N, Bensaid M, Silve S, Carayon P, Davi D, Galiegue S, Bourrie B, Guillemot JC, Ferrera P, Loison G, Maffrand JP, Le Fur G and Casellas P (1997) Purification and characterization of the human SR 31747A-binding protein. A nuclear membrane protein related to yeast sterol isomerase. *J Biol Chem* **272**: 27107–27115

Johnston SRD, MacLean KA, Sacks NPM, Salter J, Smith IE and Dowsett M (1994) Modulation of Bcl-2 and Ki-67 expression in estrogen receptor-positive human breast cancer by tamoxifen. *Eur J Cancer* **30A**: 1663–1669

Korsmeyer SJ (1992) Bcl-2 initiates a new category of oncogenes: regulators of cell death. *Blood* **80**: 879–886

Labit-Le Bouteiller C, Jamme MF, David M, Silve S, Lanau C, Dhers C, Picard C, Rahier A, Taton M, Loison G, Caput D, Ferrara P and Lupker (1998) Antiproliferative effects of SR31747A in animal cell lines are mediated by inhibition of cholesterol biosynthesis at the sterol isomerase step. *Eur J Biochem* **256**: 342–349

Leek RD, Kaklamanis L, Pezzella F, Gatter KC and Harris AL (1994) Bcl-2 in normal breast and carcinoma with estrogen receptor-positive, epidermal growth factor receptor-negative tumors and in situ cancer. *Br J Cancer* **69**: 135–139

Paul R, Silve S, De Nys N, Dupuy PH, Labit-Lebouteiller C, Rosenfeld J, Ferrara P, Le Fur G, Casellas P and Loison G (1998) Both the immunosuppressant SR31747 and the anti-estrogen tamoxifen bind to an emopamil-insensitive site of mammalian $\Delta^8$-$\Delta^7$ sterol isomerase. *J Pharmacol Exp Ther* **285**: 1296–1302

Seth P, Leibach FH and Ganapathy V (1997) Cloning and structural analysis of the cDNA and the gene encoding the murine type 1 sigma receptor. *Biochem Biophys Res Commun* **241**: 535–540

Mink D, Tngelen BV, Villena-Heinsen C, Heiss C and Schmidt W (1994) Breast cancer and prognostic factors. Tumor size, degree of differentiation, proliferation kinetics and expression of steroid hormone receptors. *Eur J Gynaecol Oncol* **6**: 424–436

Pichon MF, Broet P, Magdelenat H, Delarue JC, Spyratos F, Basuyau JP, Saez S, Rallet A, Courriere P, Millon R and Asselain B (1996) Prognostic value of steroid receptors after long-term follow-up of 2257 operable breast cancers. *Br J Cancer* **73**: 1545–1551

Robertson JF (1996) Oestrogen receptor: a stable phenotype in breast cancer. *Br J Cancer* **73**: 5–12

Silve S, Leplatois P, Josse A, Dupuy PH, Lanau C, Kaghad M, Dhers C, Picard C, Rahier A, Taton M, Le Fur G, Caput D, Ferrara P and Loison G (1996) The immunosuppressant SR 31747 blocks cell proliferation by inhibing a steroid isomerase in *Saccharomyces cerevisiae*. *Mol Cell Biol* **16**: 2719–2727

Van Slooten HJ, van de Vijver MJ, van de Velde CJ and van Dierendonck JH (1998) Loss of Bcl-2 in invasive breast cancer is associated with high rates of cell death, but also with increased proliferative activity. *Br J Cancer* **77**: 789–796

Su TP (1991) σ receptors: putative links between nervous, endocrine and immune systems. *Eur J Biochem* **200**: 633–642

 *© 2000 Cancer Research Campaign*
